# Comparative Phytochemical Profiles and Bioactivity–Constituent Relationships in Seven *Pandanus* Species From Vietnam

**DOI:** 10.1155/jamc/6373690

**Published:** 2026-10-17

**Authors:** Do Hoang Giang, Le Thanh Huong, Hoang Thuy Duong, Nguyen Thu Uyen, Nguyen Tien Dat, Nguyen Hai Dang

**Affiliations:** ^1^ University of Science and Technology of Hanoi Vietnam Academy of Science and Technology (VAST) 18 Hoang Quoc Viet, Nghia Do Hanoi 10000 Vietnam usth.edu.vn; ^2^ High Technology Innovation Center VAST 18-Hoang Quoc Viet, Nghia Do Hanoi 10000 Vietnam vast.ac.vn

**Keywords:** *α*-amylase inhibition, antioxidant activity, *Pandanus*, partial least squares regression (PLSR), phytochemical profiling, Spearman’s correlation

## Abstract

The genus *Pandanus* comprises numerous tropical plant species that are widely used in traditional medicine and local diets, yet comprehensive comparative studies linking their phytochemical composition to biological activities remain limited, particularly for underexplored species. In this study, forty‐five methanolic extracts obtained from seven *Pandanus* species (*Pandanus tectorius, Pandanus amaryllifolius, Pandanus odoratissimus, Pandanus affinis, Pandanus nanofrutex, Pandanus humilis,* and *Pandanus tonkinensis*) collected in Vietnam were systematically evaluated for their phytochemical profiles and bioactivities. Total phenolic (TPC), flavonoid (TFC), saponin (TSC), and alkaloid (TAC) contents were quantified, and antioxidant activities (DPPH and hydroxyl radical scavenging) together with *α*‐amylase inhibitory effects were assessed. Pronounced interspecific variation, together with selected exploratory organ‐related patterns, was observed, with *P. tectorius* exhibiting consistently high phenolic, flavonoid, and saponin contents and the strongest antioxidant activities, while *P. amaryllifolius* was distinguished by exceptionally high alkaloid levels. To elucidate the relationships between chemical constituents and bioactivities, partial least squares regression (PLSR) and Spearman’s correlation analyses were applied. The PLSR models showed strong explanatory and predictive performance (*Q* ^2^ = 0.889), indicating that phenolic content showed the strongest association with free radical scavenging activities, whereas saponin content was most strongly associated with *α*‐amylase inhibition. These patterns were consistent with the Spearman correlation analysis and the PLS biplot. Overall, this study provides an integrated phytochemical–bioactivity framework for *Pandanus* species, highlights distinct associations between metabolite classes and the measured bioactivities, and identifies underexplored taxa as promising candidates for future phytochemical and pharmacological investigations.

## 1. Introduction

The genus *Pandanus* (Pandanaceae) comprises more than 700 species of tropical and subtropical plants widely distributed across Asia, Africa, and the Pacific islands [1]. Members of this genus are readily recognized by their spiral arrangement of long, narrow leaves and the presence of aerial prop roots, adaptations that enable growth in coastal, sandy, and humid environments. *Pandanus* species play important ecological roles in coastal stabilization and habitat formation and are deeply embedded in local cultures, where their leaves, stems, and roots are utilized for weaving, construction, food flavoring, and traditional medicine [1]. In Vietnam, approximately ten *Pandanus* species have been documented, including *Pandanus tectorius*, *Pandanus amaryllifolius*, *Pandanus odoratissimus*, *Pandanus tonkinensis*, *Pandanus affinis,* and *Pandanus nanofrutex* [2]. Several species are valued for their aromatic leaves or inflorescences and are used in culinary, cosmetic, and medicinal practices, particularly for treating urinary, hepatic, and inflammatory conditions [2, 3]. Despite their broad distribution and ethnobotanical relevance, systematic phytochemical and bioactivity studies on many Vietnamese *Pandanus* species remain limited, highlighting the need for further investigation into their chemical constituents and biological potential.

Previous studies have demonstrated that plants of the genus *Pandanus* are rich sources of diverse bioactive constituents, including lignans [4–6], flavonoids [7], saponins [8, 9], alkaloids [10–14], and benzofuran derivatives [15, 16]. These compounds, as well as crude and enriched extracts from *Pandanus* species, have been reported to exhibit a wide range of biological activities, such as antioxidant [17], anti‐inflammatory [18, 19], cytotoxic [20, 21], and antibacterial effects [22, 23]. However, despite the considerable taxonomic diversity of the genus, phytochemical and bioactivity investigations have largely focused on a limited number of species, notably *P. tectorius*, *P. amaryllifolius*, and *P. odoratissimus* [4–6, 24, 25]. In contrast, other species, such as *P. affinis*, *P. nanofrutex*, and *P. humilis*, remain poorly studied, resulting in a significant gap in current scientific knowledge.

In this study, we systematically investigated and quantified key phytochemical constituents and evaluated the antioxidant and *α*‐amylase inhibitory activities of 45 plant samples representing seven *Pandanus* species, including *P. tectorius*, *P. amaryllifolius*, *P. odoratissimus*, *P. affinis*, *P. nanofrutex*, *P. humilis*, and *P. tonkinensis*. Furthermore, the relationships between major phytochemical groups and biological activities were examined using partial least squares (PLS) regression analysis to examine statistical associations between the major phytochemical groups and the measured bioactivities. This study provides new insights into the phytochemical composition and bioactivity–constituent relationships of underexplored *Pandanus* species, thereby contributing to their rational utilization and future functional or pharmaceutical applications.

## 2. Materials and Methods

### 2.1. Plant Materials

A total of 45 *Pandanus* samples representing seven species (*P. tectorius, P. amaryllifolius, P. odoratissimus, P. affinis, P. nanofrutex, P. humilis,* and *P. tonkinensis*) were collected from various locations in Vietnam between February 2020 and October 2024. Different plant parts, including leaves, stems, and fruits, were sampled to ensure representative chemical diversity. Detailed information on the number of samples per species, specific collection sites, and geographical coordinates of sampling locations is presented in the Supporting Information (Table S1). All plant materials were taxonomically identified by Dr. Bui Van Thanh (Institute of Biology, Vietnam Academy of Science and Technology). Voucher specimens of the collected plants were deposited at the High Technology Innovation Center, Vietnam Academy of Science and Technology. Voucher specimen accession numbers (voucher IDs) for each collected specimen are provided in the Supporting Information (Table S1).

### 2.2. Sample Preparation

To prepare the extracts for chemical analysis and bioassays, 50 g of each sample was extracted three times with 1 L of methanol in a sonication bath at 60°C for 30 min. The solution was filtered and evaporated to yield the total extract.

### 2.3. Chemical Quantitation and Bioassays

Total phenolic (TPC), total flavonoid (TFC), total alkaloid (TAC), and total saponin (TSC) contents, antioxidant effects, and *α*‐amylase inhibition of *Pandanus* extracts were evaluated using the protocols as shown in the Supporting Information.

### 2.4. Statistical Methods

All quantitative data are expressed as mean ± standard deviation (SD) based on triplicate measurements. Because the phytochemical and bioactivity data did not consistently meet the assumptions of normality and homogeneity of variance, nonparametric statistical approaches were applied. Interspecific and interorgan differences in phytochemical contents (TPC, TFC, TSC, and TAC) and biological activities were evaluated using permutation‐based Kruskal–Wallis tests with 10,000 resamplings. When differences were significant, pairwise comparisons were conducted using permutation‐based rank tests, and *p* values were adjusted for multiple testing using both the Holm method, which controls the familywise error rate, and the Benjamini–Hochberg procedure, which controls the false discovery rate. A significance threshold of *p* < 0.05 was applied throughout.

To investigate the relationships between phytochemical composition and biological activities, partial least squares regression (PLSR) was employed. PLSR was chosen because the predictor variables (TPC, TFC, TSC, and TAC) were highly correlated, which could compromise conventional multiple linear regression. In the PLSR models, the phytochemical parameters were used as predictor variables, while the bioactivity parameters served as response variables. Inhibition values measured at 50 μg/mL for the DPPH and hydroxyl radical scavenging assays and at 200 μg/mL for the *α*‐amylase inhibition assay were selected for modeling. All variables were mean‐centered and autoscaled before analysis. Model validation was performed using fivefold cross‐validation, in which the observations were randomly divided into five segments. A fixed random seed was used to ensure reproducibility of the cross‐validation procedure. The optimal number of latent components was selected based on the highest mean cross‐validated coefficient of determination across the three response variables. Model performance was evaluated using the coefficients of determination for calibration (*R* ^2^) and cross‐validation (*Q* ^2^), together with the root mean square errors of prediction for calibration and cross‐validation (RMSEP). All statistical analyses were performed using R software.

## 3. Results and Discussion

### 3.1. Phytochemical Investigation of the Plant Samples From the Genus *Pandanus*

The phytochemical investigation of 45 methanolic extracts obtained from seven *Pandanus* species revealed pronounced interspecific variation, together with selected organ‐related patterns, in TPC, TFC, TSC, and TAC. These patterns are visualized using sample‐level faceted dot plots (Figure 1), in which each point represents an individual extract, colors distinguish species, and point shapes distinguish plant organs. Detailed quantitative values for individual samples, including sample codes and plant parts, are presented in Table S2.

**FIGURE 1:**
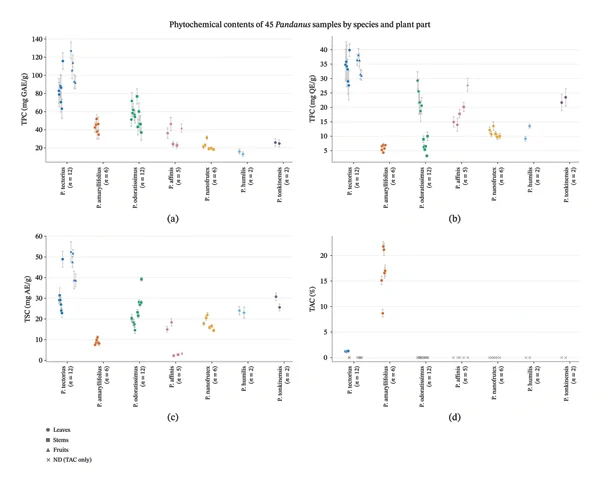
Sample‐level faceted dot plots showing the distribution of major phytochemical groups in 45 *Pandanus* extracts: (a) Total phenolic content (TPC, mg GAE/g), (b) total flavonoid content (TFC, mg QE/g), (c) total saponin content (TSC, mg AE/g), and (d) total alkaloid content (TAC, %). Colors distinguish species, whereas point shapes distinguish plant organs. Points and error bars represent the mean ± SD of triplicate measurements. Crosses indicate TAC values below the limit of detection.

As illustrated in Figure 1a, TPC varied widely among species, with *P. tectorius* clearly standing out as the phenolic‐rich taxon. Across all samples, TPC values ranged from approximately 13 to 127 mg GAE/g. Fruits of *P. tectorius* exhibited particularly high phenolic contents, exceeding 90 mg GAE/g and reaching up to 127 mg GAE/g, while leaves also showed consistently elevated levels. Permutation‐based Kruskal–Wallis testing confirmed highly significant interspecific differences in TPC (*H* = 38.79, *p* < 0.001). Pairwise post hoc comparisons demonstrated that *P. tectorius* possessed significantly higher phenolic contents than all other species after both Holm and Benjamini–Hochberg adjustments (all adjusted *p* < 0.01), establishing it as the dominant phenolic accumulator within the genus. Additional contrasts indicated that *P. odoratissimus* and *P. amaryllifolius* tended to contain higher phenolic levels than *P. affinis* and *P. nanofrutex*, although several of these differences were significant only under BH adjustment, reflecting moderate separation that does not withstand stricter familywise error control. Several species pairs, including *P. affinis* vs. *P. humilis* and *P. nanofrutex* vs. *P. tonkinensis*, have no significant differences under either correction method.

A broadly similar pattern was observed for flavonoids (Figure 1b), although with clearer differentiation of certain taxa. TFC values across the dataset ranged from approximately 4 to 40 mg QE/g. *P. tectorius* again exhibited the highest flavonoid levels, particularly in fruits and leaves, and differed significantly from most other species according to permutation‐based Kruskal–Wallis analysis (*H* = 32.65, *p* < 0.001). Post hoc tests confirmed that *P. tectorius* contained significantly higher flavonoid levels than *P. amaryllifolius*, *P. humilis*, and *P. nanofrutex* after Holm adjustment, while *P. amaryllifolius* was consistently flavonoid‐poor, with values largely below 7 mg QE/g. Intermediate and partially overlapping flavonoid levels were observed for *P. odoratissimus*, *P. affinis*, and *P. tonkinensis*. Several contrasts among these intermediate taxa reached nominal significance but were not retained after Holm correction, again illustrating the sensitivity of the observed differences to the chosen multiple testing adjustment.

Saponin contents exhibited a more heterogeneous distribution across species (Figure 1c), with values ranging from approximately 2 to 53 mg AE/g. Interspecific differences in TSC were highly significant (*H* = 34.05, *p* < 0.001). *P. tectorius* again showed the highest saponin levels, particularly in fruits, which frequently exceeded 40 mg AE/g, while leaves generally ranged between 23 and 31 mg AE/g. Pairwise comparisons confirmed that *P. tectorius* differed significantly from *P. affinis*, *P. amaryllifolius*, *P. nanofrutex*, and *P. odoratissimus* under Holm adjustment. *P. tonkinensis* also exhibited relatively high and stable saponin contents (approximately 26–31 mg AE/g), and although some comparisons with low‐saponin species reached significance under BH adjustment, these differences were not always retained under Holm correction. *P. odoratissimus*, *P. nanofrutex*, and *P. humilis* formed an intermediate group with overlapping saponin ranges, whereas *P. affinis* and *P. amaryllifolius* generally displayed the lowest saponin contents.

In contrast to the other phytochemical classes, alkaloids showed a strongly polarized distribution pattern (Figure 1d). TAC values ranged from nondetectable levels in most species to more than 20% in *P. amaryllifolius*. Permutation‐based Kruskal–Wallis analysis confirmed highly significant interspecific variation (*H* = 32.66, *p* < 0.001), driven almost entirely by the exceptional alkaloid accumulation in *P. amaryllifolius*. Pairwise comparisons demonstrated that this species differed significantly from *P. affinis*, *P. nanofrutex*, *P. odoratissimus*, and *P. tectorius* under both Holm and BH adjustments, whereas all other species showed uniformly low or undetectable alkaloid levels and did not differ significantly from one another. This highly species‐specific accumulation pattern is consistent with previous reports indicating that alkaloids are a characteristic and abundant phytochemical class in *P. amaryllifolius*, while being rare or absent in most other *Pandanus* species [10, 11, 24, 25].

Taken together, the sample‐level visualization and nonparametric analyses demonstrate that phytochemical variation in the investigated *Pandanus* samples was primarily associated with species identity, although selected organ‐related patterns were also evident. Most within‐species organ comparisons were not statistically significant after correction for multiple testing, with the notable exceptions of the higher TFC in leaves and higher TSC in stems of *P. odoratissimus*. *P. tectorius* was characterized by generally high phenolic, flavonoid, and saponin contents across both leaves and fruits, whereas *P. amaryllifolius* exhibited a distinctive alkaloid‐rich profile. *P. tonkinensis* was notable for its relatively high leaf saponin contents. Because the numbers of samples and organs were unequal among species, particularly for *P. affinis*, *P. humilis*, and *P. tonkinensis*, these organ‐level observations should be regarded as exploratory rather than conclusive. Nevertheless, the sample‐ and organ‐resolved profiles provide a clearer basis for interpreting the subsequent bioactivity screening and multivariate analyses.

### 3.2. Free Radical Scavenging and *α*‐Amylase Inhibitory Activities of the *Pandanus* Plants’ Extracts

In the DPPH assays, *P. tectorius* extracts consistently displayed the strongest activity among the investigated taxa. At 20 μg/mL, inhibition ranged from 18% to 35% and increased to 56%–76% at 50 μg/mL, approaching the activity of ascorbic acid (88%). Ptec04 and Ptec08 were the most active extracts, exceeding 75% inhibition at 50 μg/mL. Interspecific comparisons showed that *P. tectorius* differed significantly from almost all other species at both concentrations (*p* < 0.01). *P. odoratissimus* showed intermediate activity, with inhibition values of 13%–29% at 20 μg/mL and 36%–60% at 50 μg/mL, whereas *P. amaryllifolius* generally showed weaker responses. Extracts of *P. affinis*, *P. nanofrutex*, *P. humilis*, and *P. tonkinensis* were generally the least active and showed no significant differences from one another (*p* > 0.05). A similar concentration‐dependent pattern was observed in the hydroxyl radical assay. *P. tectorius* again showed the highest activity, increasing from 22%–36% at 20 μg/mL to 56%–74% at 50 μg/mL, followed by *P. odoratissimus*. The remaining species exhibited weak‐to‐moderate hydroxyl radical scavenging and were generally less active than *P. tectorius* and *P. odoratissimus*.

Within individual species, samples from different plant organs generally showed overlapping activity ranges, and no consistent organ‐dependent pattern was observed across the two radical scavenging assays. Leaves and fruits of *P. tectorius*, as well as leaves and stems of *P. odoratissimus*, included samples with comparable activities, although some individual fruit or stem extracts showed relatively high responses. Similarly, the leaf and stem extracts of *P. affinis* and *P. nanofrutex* did not display a uniform separation according to plant part. These findings suggest that the differences in radical scavenging activity were more apparent among species than among organs within the same species, although the unequal numbers of samples and organs limit broader interpretation.

For *α*‐amylase inhibition, a similar concentration‐dependent trend was observed. At 100 μg/mL, inhibition among *P. tectorius* extracts ranged from 16% to 28% and increased to 34%–64% at 200 μg/mL, with Ptec07 and Ptec08 showing the strongest responses. *P. tectorius* exhibited significantly higher inhibition than most other species at both concentrations (*p* < 0.01), while *P. odoratissimus* showed moderate activity. *P. tonkinensis*, despite its weak radical scavenging activity, showed appreciable *α*‐amylase inhibition at 200 μg/mL (32%–46%) and did not differ significantly from *P. odoratissimus* or *P. tectorius* (*p* > 0.1). Conversely, *P. amaryllifolius*, *P. affinis*, *P. nanofrutex*, and *P. humilis* generally showed weak enzyme inhibition, with no significant differences among these species (*p* > 0.05). As in the radical scavenging assays, *α*‐amylase inhibition did not show a consistent separation according to plant organ within the species represented by multiple organs. None of the extracts matched the activity of the corresponding positive controls. Nevertheless, the observed responses support further phytochemical investigation of *P. tectorius* and selected *P. odoratissimus* and *P. tonkinensis* extracts for constituents associated with antioxidant and *α*‐amylase‐inhibitory activities.

IC_50_ values are commonly used to compare bioactivity potency; however, they could not be reliably determined for several extracts because inhibition remained too low to reach 50% within the experimentally appropriate concentration range. Restricting the multivariate analysis to samples with measurable IC_50_ values would have excluded the weaker extracts and disrupted the continuity and comparability of the dataset. Therefore, inhibition percentages measured at fixed screening concentrations were used as the response variables in the PLSR analysis. Specifically, the values obtained at 50 μg/mL for DPPH and hydroxyl radical scavenging and at 200 μg/mL for *α*‐amylase inhibition were selected because these concentrations provided measurable responses across the complete set of extracts while retaining weakly active samples in the analysis. These fixed‐concentration data should be interpreted as comparative screening responses rather than direct estimates of potency (Table 1).

**TABLE 1:** Screening results of radical scavenging (DPPH and hydroxyl) and *α*‐amylase inhibitory activities of extracts from *Pandanus* species.

No.	Type	Part	Code	DPPH scavenging effect, %I		Hydroxyl, %I		*α*‐amylase inhibition, %I	
				20 μg/mL	50 μg/mL	20 μg/mL	50 μg/mL	100 μg/mL	200 μg/mL
1	*P. tectorius*	Leaves	Ptec01	29.28 ± 1.03	62.49 ± 2.20	31.24 ± 2.48	63.85 ± 4.14	19.10 ± 2.08	54.87 ± 6.49
2	*P. tectorius*	Leaves	Ptec02	23.10 ± 2.45	59.90 ± 7.16	30.50 ± 1.66	61.55 ± 5.15	20.77 ± 2.18	53.98 ± 5.32
3	*P. tectorius*	Leaves	Ptec03	32.40 ± 3.85	65.56 ± 6.24	25.22 ± 2.64	68.87 ± 3.11	28.16 ± 0.94	57.64 ± 2.52
4	*P. tectorius*	Leaves	Ptec04	26.96 ± 2.71	76.11 ± 7.98	34.97 ± 3.98	73.41 ± 3.72	16.09 ± 1.72	41.21 ± 1.64
5	*P. tectorius*	Leaves	Ptec05	21.95 ± 1.15	58.51 ± 5.44	23.37 ± 2.78	60.50 ± 6.97	16.63 ± 1.53	38.10 ± 1.92
6	*P. tectorius*	Leaves	Ptec06	18.35 ± 1.93	56.12 ± 2.88	24.23 ± 0.98	55.50 ± 3.10	16.84 ± 0.88	33.87 ± 3.41
7	*P. tectorius*	Leaves	Ptec07	22.79 ± 1.25	63.10 ± 4.73	22.32 ± 2.07	68.70 ± 7.47	26.94 ± 2.63	62.43 ± 5.89
8	*P. tectorius*	Fruits	Ptec08	35.32 ± 3.27	76.25 ± 8.09	33.05 ± 3.92	73.51 ± 6.03	22.71 ± 2.50	63.51 ± 4.44
9	*P. tectorius*	Fruits	Ptec09	23.72 ± 1.81	70.02 ± 6.72	25.42 ± 2.58	71.76 ± 7.29	27.39 ± 2.85	58.43 ± 2.11
10	*P. tectorius*	Fruits	Ptec10	32.23 ± 3.08	72.68 ± 3.61	31.15 ± 1.04	72.94 ± 6.09	18.99 ± 0.70	56.82 ± 3.81
11	*P. tectorius*	Fruits	Ptec11	29.57 ± 2.46	70.58 ± 8.14	35.94 ± 4.21	72.23 ± 7.68	22.66 ± 1.92	59.31 ± 5.93
12	*P. tectorius*	Fruits	Ptec12	22.70 ± 1.96	68.91 ± 2.90	29.17 ± 2.17	70.78 ± 7.05	21.88 ± 2.17	58.32 ± 6.58
13	*P. amaryllifolius*	Leaves	Pama01	16.27 ± 1.76	46.07 ± 4.12	11.95 ± 0.74	32.12 ± 2.91	6.27 ± 0.35	12.78 ± 1.04
14	*P. amaryllifolius*	Leaves	Pama02	20.98 ± 2.48	46.92 ± 2.16	10.97 ± 1.26	34.00 ± 1.43	5.02 ± 0.30	13.78 ± 1.30
15	*P. amaryllifolius*	Leaves	Pama03	19.23 ± 1.90	51.41 ± 4.04	22.29 ± 1.03	46.63 ± 5.61	6.97 ± 0.31	14.87 ± 1.72
16	*P. amaryllifolius*	Leaves	Pama04	13.99 ± 0.51	37.31 ± 1.24	15.49 ± 1.45	31.60 ± 2.29	7.04 ± 0.29	15.32 ± 0.58
17	*P. amaryllifolius*	Leaves	Pama05	16.88 ± 1.86	48.73 ± 2.07	18.65 ± 1.58	42.66 ± 1.73	5.21 ± 0.56	13.51 ± 1.52
18	*P. amaryllifolius*	Leaves	Pama06	16.41 ± 1.42	33.47 ± 2.33	12.47 ± 0.42	30.37 ± 3.01	6.49 ± 0.22	13.06 ± 0.58
19	*P. odoratissimus*	Leaves	Podo01	20.36 ± 2.42	50.74 ± 2.18	15.94 ± 1.17	45.02 ± 4.88	9.84 ± 0.58	24.40 ± 2.73
20	*P. odoratissimus*	Leaves	Podo02	25.89 ± 2.88	58.42 ± 7.02	25.34 ± 1.51	59.00 ± 3.40	15.30 ± 0.64	33.48 ± 3.31
21	*P. odoratissimus*	Leaves	Podo03	21.27 ± 1.50	54.37 ± 6.42	16.73 ± 1.53	52.90 ± 3.42	8.97 ± 0.59	24.98 ± 2.05
22	*P. odoratissimus*	Leaves	Podo04	21.26 ± 2.57	57.76 ± 3.20	24.95 ± 2.68	58.79 ± 3.69	9.08 ± 0.46	25.81 ± 2.36
23	*P. odoratissimus*	Leaves	Podo05	17.74 ± 0.92	52.93 ± 4.71	23.68 ± 0.89	50.77 ± 3.88	10.68 ± 0.56	23.62 ± 1.46
24	*P. odoratissimus*	Leaves	Podo06	18.36 ± 1.78	52.00 ± 5.34	24.72 ± 2.32	50.17 ± 4.04	9.94 ± 1.17	22.05 ± 0.95
25	*P. odoratissimus*	Stems	Podo07	29.19 ± 3.19	60.01 ± 6.64	29.11 ± 2.53	62.23 ± 7.15	18.61 ± 2.24	38.86 ± 1.47
26	*P. odoratissimus*	Stems	Podo08	19.89 ± 1.14	48.20 ± 2.47	15.24 ± 1.25	31.78 ± 2.02	8.24 ± 0.86	24.87 ± 1.34
27	*P. odoratissimus*	Stems	Podo09	28.68 ± 2.10	58.56 ± 2.32	27.92 ± 1.73	60.63 ± 2.43	23.06 ± 1.43	49.70 ± 4.81
28	*P. odoratissimus*	Stems	Podo10	17.28 ± 0.64	50.61 ± 3.47	19.89 ± 1.97	44.51 ± 2.67	20.04 ± 2.36	41.18 ± 4.92
29	*P. odoratissimus*	Stems	Podo11	15.08 ± 1.15	47.22 ± 3.35	17.35 ± 1.77	37.31 ± 2.82	14.50 ± 1.11	42.90 ± 2.02
30	*P. odoratissimus*	Stems	Podo12	13.32 ± 0.46	36.45 ± 3.19	14.94 ± 1.66	31.40 ± 3.13	21.21 ± 1.65	45.30 ± 2.92
31	*P. affinis*	Leaves	Paff01	12.63 ± 0.55	39.62 ± 2.91	14.91 ± 1.41	30.34 ± 2.28	6.72 ± 0.31	21.05 ± 2.43
32	*P. affinis*	Leaves	Paff02	15.32 ± 1.21	48.59 ± 5.83	15.63 ± 1.21	41.63 ± 2.83	10.00 ± 0.80	22.53 ± 2.28
33	*P. affinis*	Stems	Paff03	13.87 ± 1.30	28.67 ± 2.65	12.26 ± 1.12	25.80 ± 1.41	2.20 ± 0.11	5.43 ± 0.57
34	*P. affinis*	Stems	Paff04	8.74 ± 0.56	18.42 ± 2.00	10.60 ± 0.57	21.46 ± 1.04	3.07 ± 0.12	6.40 ± 0.21
35	*P. affinis*	Fruits	Paff05	21.56 ± 2.22	44.36 ± 4.59	12.38 ± 0.45	32.11 ± 3.07	4.18 ± 0.38	8.62 ± 0.41
36	*P. nanofrutex*	Leaves	Pnano01	7.33 ± 0.33	16.35 ± 1.25	10.05 ± 0.64	20.29 ± 1.88	8.24 ± 0.30	18.62 ± 0.80
37	*P. nanofrutex*	Leaves	Pnano02	7.08 ± 0.34	22.73 ± 1.47	8.90 ± 0.89	24.80 ± 2.02	11.36 ± 0.42	24.31 ± 1.59
38	*P. nanofrutex*	Leaves	Pnano03	11.93 ± 0.98	25.31 ± 1.11	9.65 ± 0.68	28.52 ± 1.77	11.82 ± 1.03	26.39 ± 1.38
39	*P. nanofrutex*	Stems	Pnano04	10.00 ± 0.90	26.05 ± 2.31	9.18 ± 0.56	26.98 ± 2.97	11.45 ± 1.27	23.10 ± 0.91
40	*P. nanofrutex*	Stems	Pnano05	9.28 ± 0.72	28.29 ± 3.27	8.39 ± 0.57	18.21 ± 1.73	8.32 ± 0.60	21.30 ± 1.99
41	*P. nanofrutex*	Stems	Pnano06	8.61 ± 0.69	17.81 ± 2.02	7.34 ± 0.38	16.81 ± 1.26	9.43 ± 0.87	21.50 ± 0.83
42	*P. humilis*	Leaves	Phum01	7.70 ± 0.80	16.83 ± 1.53	9.63 ± 0.66	23.91 ± 1.91	11.08 ± 1.09	34.40 ± 2.78
43	*P. humilis*	Leaves	Phum02	6.49 ± 0.66	14.05 ± 1.10	7.01 ± 0.28	20.73 ± 1.96	11.16 ± 1.12	27.55 ± 2.47
44	*P. tonkinensis*	Leaves	Ptonk01	8.39 ± 0.57	22.43 ± 2.02	12.93 ± 1.28	28.44 ± 1.79	21.16 ± 1.91	45.73 ± 1.59
45	*P. tonkinensis*	Leaves	Ptonk02	7.74 ± 0.43	22.43 ± 2.22	10.57 ± 0.41	26.74 ± 1.04	12.39 ± 1.46	31.85 ± 1.85
Control	Ascorbic acid^∗^			38.49 ± 2.60	88.23 ± 3.55	—	—	—	—
Control	Catechin^∗∗^			—	—	32.44 ± 1.77	79.44 ± 3.78	—	—
Control	Acarbose^∗∗∗^			—	—	—	—	49.31 ± 4.14	81.17 ± 7.78

*Note:* Values are presented as mean ± SD from three replicate measurements. Extract concentrations were 20 and 50 μg/mL for radical scavenging assays and 100 and 200 μg/mL for *α*‐amylase inhibition. Ascorbic acid, catechin, and acarbose were used as positive controls (^∗^, ^∗∗^, and ^∗∗∗^, respectively).

### 3.3. Correlation Between Chemical Components and Free Radical Scavenging and *α*‐Amylase Inhibitory Activities of the *Pandanus* Plants’ Extracts

The radical scavenging assays revealed marked variation among the investigated *Pandanus* extracts. Samples of *P. tectorius* generally showed the strongest and most consistent DPPH and hydroxyl radical scavenging activities, followed by selected samples of *P. odoratissimus*, whereas most extracts from the remaining species were comparatively less active. However, the individual assays alone could not identify the phytochemical variables associated with these responses. PLSR was therefore applied to examine the relationships between four phytochemical parameters—TPC, TFC, TSC, and TAC—and three measured bioactivities, namely DPPH radical scavenging, hydroxyl radical scavenging, and *α*‐amylase inhibition.

The PLSR models showed strong explanatory and cross‐validated performance within the present dataset. At the selected optimum of four components, the cumulative explained variance of the *X*‐matrix was *R* ^2^ *X*_train_cum = 1.00, and the mean cross‐validated predictive ability was *Q* ^2^_mean = 0.889 (Table S3). For the individual responses, *R* ^2^ *Y*_train values ranged from 0.898 to 0.917, while *Q* ^2^_CV values ranged from 0.879 to 0.902 (Table S3). The first two latent variables accounted for 85.56% of the total *X*‐variance, with PLS1 and PLS2 explaining 63.15% and 22.41%, respectively (Table S4). The biplot based on these two components therefore captured most of the variation in the measured phytochemical variables and provided an informative representation of the relationships among samples, constituent classes, and bioactivities (Figure 2).

**FIGURE 2:**
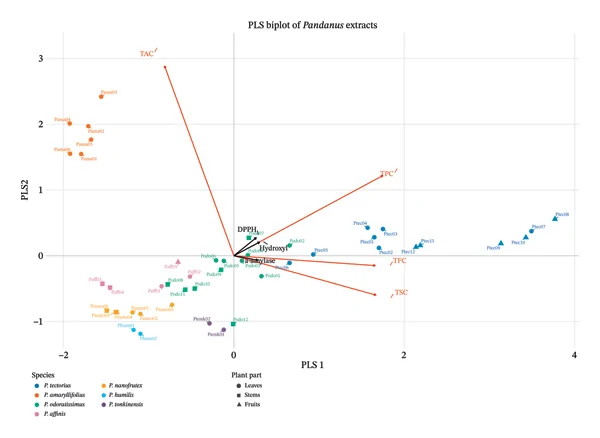
PLS biplot of *Pandanus* extracts showing the relationships between phytochemical parameters (TPC, TFC, TSC, and TAC) and bioactivities (DPPH, hydroxyl radical scavenging at 50 μg/mL, and α‐amylase inhibition at 200 μg/mL).

The biplot, in which colors distinguish species and symbols distinguish plant organs, indicates that the distribution of the samples was primarily associated with species identity rather than plant part. Samples of *P. tectorius* were predominantly positioned on the positive side of PLS1, corresponding to their generally high TPC, TFC, and TSC values and comparatively strong bioactivities. In contrast, *P. amaryllifolius* samples formed a distinct cluster on the negative side of PLS1 and the positive side of PLS2, in the direction associated with TAC. Samples of *P. odoratissimus* occupied an intermediate region near the center of the biplot, while *P. nanofrutex* and *P. humilis* were mainly distributed in the negative region of both PLS1 and PLS2. Samples of *P. affinis* and *P. tonkinensis* were also located predominantly on the negative side of PLS1 but showed partial overlap with other taxa. These distributions suggest that sample differentiation in the first two latent dimensions was more strongly associated with species‐specific phytochemical profiles than with plant organ identity.

In contrast, no consistent separation according to plant organ was evident across the complete dataset. Leaf, stem, and fruit symbols did not form discrete organ‐specific clusters, and samples derived from different organs frequently overlapped within the same regions of the biplot. Within *P. tectorius*, most fruit samples were positioned further along the positive direction of PLS1 than most leaf samples, consistent with the relatively high phenolic and saponin contents of several fruits. Nevertheless, the leaf sample Ptec07 occupied the same positive‐PLS1 region as the fruit samples, indicating that the observed distribution did not represent a complete leaf–fruit separation. Similarly, the leaf and stem samples of *P. odoratissimus* and *P. nanofrutex* were interspersed, while the leaf, stem, and fruit samples of *P. affinis* did not form distinct organ‐specific groups. Thus, although some within‐species organ‐related tendencies may be present, the first two PLS components did not provide evidence of a general or consistent classification according to plant part.

The absence of clear organ‐level clustering should be interpreted cautiously. Plant organs were not equally represented among species: *P. amaryllifolius*, *P. humilis*, and *P. tonkinensis* were represented only by leaves, whereas only one fruit sample was available for *P. affinis*. Moreover, the PLSR model was developed to describe phytochemical–bioactivity relationships rather than to perform supervised classification of plant organs. Therefore, the biplot indicates that organ identity was not a dominant source of separation in the first two latent dimensions, but it does not exclude more subtle or species‐specific organ effects.

Among the phytochemical variables, TPC had the highest VIP score (VIP = 1.27; Table S6), identifying it as the most influential predictor in the combined PLSR models. Its position in the biplot, together with the distribution of the phenolic‐rich and strongly active *P. tectorius* samples, was consistent with a close association between phenolic content and radical scavenging responses. TSC also had a VIP score above the conventional threshold of one (VIP = 1.03; Table S6) and was oriented toward the region associated with *α*‐amylase inhibition, supporting an association between saponin content and this enzyme‐inhibitory response. TFC showed intermediate predictive importance, with a VIP value of 0.96, which was lower than the corresponding values of TPC and TSC in the combined model. TAC was oriented primarily along PLS2, which explained 22.41% of the X‐variance. The clear positioning of *P. amaryllifolius* samples in the TAC‐associated region reflected their high alkaloid contents and distinguished them from the other investigated species. However, TAC had a relatively low VIP score of 0.66 and showed no strong association with DPPH, hydroxyl radical scavenging, or *α*‐amylase inhibition. Thus, the distinct separation of *P. amaryllifolius* appears to reflect its alkaloid‐rich phytochemical profile rather than strong activity in the three assays examined here. This result does not imply that its alkaloids are biologically inactive. Our previous study demonstrated notable anti‐inflammatory activity of alkaloid fractions isolated from *P. amaryllifolius*, indicating that these compounds may be more relevant to biological endpoints beyond those evaluated in the present work. The PLSR models also produced low prediction errors, with RMSEP_train values of 0.052–0.053 and RMSEP_CV values of 0.057–0.060 (Table S5). These results support the internal predictive performance of the models for the three measured responses. Nevertheless, because species and plant organs were represented unequally, the PLSR findings should be interpreted as associations within the investigated sample set rather than as evidence that a particular phytochemical class directly causes the observed bioactivity.

The Spearman correlation heatmap (Figure 3) provides an integrated visualization of the relationships between major phytochemical constituents and the bioactivities of *Pandanus* extracts, thereby providing a complementary assessment that was consistent with the multivariate PLS regression analysis. Strong positive correlations were observed between TPC and both DPPH radical scavenging (*ρ* = 0.98, *p* < 0.001) and hydroxyl radical scavenging activities (*ρ* = 0.99, *p* < 0.001) (Tables S7 and S8), indicating a strong positive association between TPC and radical scavenging activity in the investigated extracts. A moderate yet statistically significant correlation was also detected between TPC and *α*‐amylase inhibitory activity (*ρ* = 0.66, *p* < 0.001), indicating a moderate positive association between TPC and *α*‐amylase inhibition. These results are in excellent agreement with the PLS biplot (Figure 2), in which TPC was strongly aligned with DPPH and OH responses along the positive direction of PLS1, as well as with its high VIP score in the PLSR model.

**FIGURE 3:**
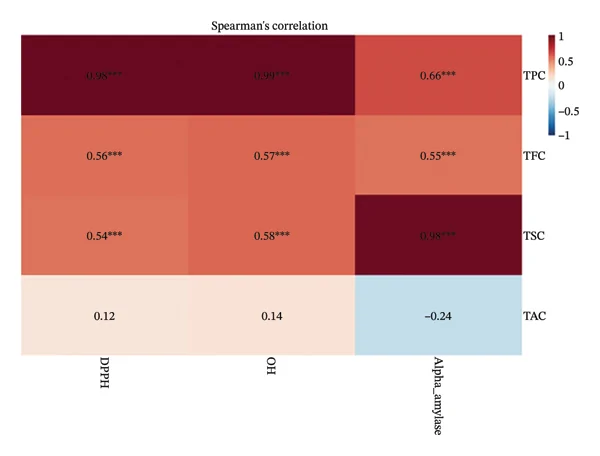
Spearman’s correlation heatmap illustrating the relationships between phytochemical constituents and bioactivities of *Pandanus* extracts. Asterisks indicate statistical significance (^∗∗∗^ *p* < 0.001).

TSC exhibited an exceptionally strong positive correlation with *α*‐amylase inhibition (*ρ* = 0.98, *p* < 0.001), while showing moderate but statistically significant correlations with DPPH and OH scavenging activities (*ρ* = 0.54–0.58, *p* < 0.001) (Figure 3; Tables S7 and S8). This correlation pattern closely mirrors the PLSR results, where TSC was positioned toward the *α*‐amylase response in the biplot (Figure 2) and displayed a VIP value exceeding unity, indicating that TSC showed the strongest association with *α*‐amylase inhibition among the investigated phytochemical parameters. TFC showed moderate and statistically significant correlations with all three bioactivities (*ρ* = 0.55–0.57, *p* < 0.001), consistent with its moderate associations and intermediate predictive importance in the PLSR model.

In contrast, TAC demonstrated weak and statistically nonsignificant correlations with DPPH and OH scavenging activities (*ρ* = 0.12–0.14, *p* > 0.05) and a weak negative association with *α*‐amylase inhibition (*ρ* = −0.24, *p* > 0.05) (Tables S7 and S8). This observation is consistent with the PLSR results, in which TAC was primarily associated with the second latent variable rather than PLS1 in the biplot (Figure 2) and exhibited a relatively low VIP score, consistent with its weak associations with the antioxidant and *α*‐amylase inhibitory activities investigated in this study. Notably, samples of *P. amaryllifolius* were clearly separated along the TAC‐associated direction in the biplot, reflecting the unique alkaloid‐rich chemical profile of this species. Although TAC showed no significant association with the bioactivities examined here, this distinct separation supports further evaluation of the alkaloid‐rich extracts of *P. amaryllifolius* using biological endpoints beyond those examined in the present study.

Overall, the agreement between the Spearman correlation analysis and PLSR indicates that phenolic content was most strongly associated with free radical scavenging activity, whereas saponin content was more closely associated with *α*‐amylase inhibition. Flavonoids showed moderate associations with all three responses, while TAC was not significantly associated with the measured activities. The revised biplot further demonstrates that sample differentiation occurred primarily at the species level. Although some local organ‐related tendencies were visible, particularly among *P. tectorius* samples, no consistent separation of leaves, stems, and fruits was observed across the investigated taxa. Accordingly, the present results support species‐level phytochemical differentiation but do not provide evidence for a general organ‐based classification in the first two PLS dimensions.

### 3.4. Discussion

The present study provides a broader comparative perspective on the genus *Pandanus* by evaluating 45 samples representing seven species under a common extraction, phytochemical quantification, and bioactivity screening framework. Previous phytochemical investigations of this genus have primarily focused on individual species, selected plant organs, or the isolation of compounds, especially from *P. tectorius*, *P. amaryllifolius*, and *P. odoratissimus* [4–17]. Consequently, direct comparison among species has remained difficult because of differences in extraction procedures, assay conditions, geographical origin, and plant material. By applying the same experimental procedures to all samples and integrating conventional phytochemical assays with PLSR and Spearman’s correlation analyses, the present study provides a systematic assessment of interspecific phytochemical variation and selected exploratory organ‐related patterns among Vietnamese *Pandanus* samples.

Among the investigated taxa, *P. tectorius* was distinguished by consistently high phenolic, flavonoid, and saponin contents and by the strongest DPPH and hydroxyl radical scavenging activities. This finding is consistent with previous chemical studies demonstrating that *P. tectorius* is a rich source of structurally diverse phenolic constituents. Phenolic compounds and flavonoids have previously been isolated from its fruits [7], together with a dihydrofurocoumarin [6] and several other secondary metabolites [8, 9]. More recently, benzofuran sesquineolignan racemates were characterized from the roots and rhizomes of this species [16], further indicating a substantial biosynthetic capacity for phenolic and lignan‐type compounds. Andriani et al. also reported that both the keys and cores of *P. tectorius* fruits contained phytochemicals with antioxidant, antibacterial, and cytotoxic properties [17]. The strong radical scavenging activities observed in the present study therefore agree with the previously reported antioxidant potential of this species, while extending these findings across a larger collection of Vietnamese samples and different plant organs. The relatively strong *α*‐amylase inhibition exhibited by *P. tectorius* is particularly noteworthy because most previous investigations of this species have emphasized antioxidant, antimicrobial, cytotoxic, or compound‐isolation studies rather than carbohydrate‐digesting enzyme inhibition [6–8, 16, 17]. Although the crude extracts remained less active than acarbose, several *P. tectorius* samples produced substantial inhibition at 200 μg/mL. These results suggest that the species may contain constituents associated with both radical scavenging and *α*‐amylase‐inhibitory responses. Nevertheless, the present in vitro findings should be regarded as evidence for prioritizing further investigation rather than as direct proof of an antidiabetic effect. Isolation of the active constituents, determination of enzyme‐inhibition kinetics, and confirmation in cellular and in vivo models will be required before any physiological relevance can be established.

The intermediate position of *P. odoratissimus* in the antioxidant assays is also consistent with previous reports. Natural products have been isolated from the fruits of this species [4], and Jong and Chau demonstrated antioxidative activities of constituents isolated from *P. odoratissimus* [15]. These earlier observations support the moderate‐to‐strong radical scavenging effects detected in several *P. odoratissimus* samples in the present study. In addition, aqueous extracts of *P. odoratissimus* roots and leaves have shown anticancer potential in both in vitro and in vivo experimental models [20, 21]. In the present study, *P. odoratissimus* was the main species showing a discernible organ‐related pattern: Leaves contained higher total flavonoid levels, whereas stems contained higher total saponin levels. Nevertheless, leaf and stem samples still showed considerable overlap in the PLS biplot and did not form completely separate multivariate clusters. Thus, the organ‐related differences in this species appear to be specific to phytochemical parameters rather than indicative of a general separation between leaves and stems. For the other species represented by more than one organ, the chemical and bioactivity profiles generally overlapped, and no consistent organ‐based differentiation was observed. Comparisons were not possible for species represented exclusively by leaves.

In contrast, *P. amaryllifolius* displayed a highly specialized phytochemical profile characterized by exceptionally high TAC but comparatively low phenolic, flavonoid, and saponin levels. This pattern is in strong agreement with the established chemistry of the species. A series of characteristic alkaloids, including pandanamine, pandamarilactonines, norpandamarilactonines, and pandamarine, have been isolated and structurally characterized from *P. amaryllifolius* [10–14, 24]. The pronounced separation of its samples along the TAC‐associated axis in the PLS biplot therefore reflects a genuine chemotaxonomic feature rather than simply analytical variation. However, the relatively weak radical scavenging and *α*‐amylase inhibitory activities of these alkaloid‐rich extracts indicate that alkaloid abundance does not necessarily translate into strong activity in the assays examined here. This interpretation is supported by the low VIP score and weak Spearman’s correlations obtained for TAC. The weak associations between TAC and the tested responses should not be interpreted as evidence that the alkaloids of *P. amaryllifolius* are biologically inactive. A recent investigation reported antimicrobial potential and antioxidant properties for extracts of this species [23], while alkaloid fractions obtained from Vietnamese *P. amaryllifolius* demonstrated anti‐inflammatory effects [26]. Thus, the chemical specialization of *P. amaryllifolius* may be more relevant to antimicrobial, anti‐inflammatory, or other biological endpoints than to direct radical scavenging or *α*‐amylase inhibition. This finding illustrates the importance of matching phytochemical classes with appropriate biological assays rather than assuming that extracts rich in secondary metabolites will display uniformly strong activity across unrelated pharmacological targets. An additional finding of interest was the comparatively high and stable saponin content of *P. tonkinensis*. Despite its weak DPPH and hydroxyl radical scavenging activities, the species showed appreciable *α*‐amylase inhibition, particularly at 200 μg/mL. Previous investigation of *P. tonkinensis* fruits identified several constituents with inhibitory effects on NO production and lipid peroxidation [9]. The present results extend the available knowledge by indicating that extracts of this species may also contain constituents affecting carbohydrate‐hydrolyzing enzymes. However, because only two *P. tonkinensis* samples were included, this apparent selectivity should be considered preliminary. Further sampling and compound‐level analysis are required to determine whether the observed *α*‐amylase inhibition represents a consistent species‐specific property. For *P. affinis*, *P. nanofrutex*, and *P. humilis*, published phytochemical and pharmacological information remains very limited. Their generally lower phenolic contents and weaker radical scavenging activities distinguish them from *P. tectorius* and *P. odoratissimus* under the present experimental conditions. Nevertheless, low activity in the DPPH, hydroxyl radical, and *α*‐amylase assays does not exclude the presence of compounds active against other biological targets.

Studies of other *Pandanus* species have demonstrated anti‐inflammatory, antinociceptive, antibacterial, and cytotoxic activities that are not necessarily predicted by simple radical scavenging assays [17, 18, 22]. The present quantitative profiles should therefore be regarded as a baseline for these underexplored taxa and as a guide for selecting complementary assays rather than as a definitive assessment of their pharmacological value. The strong correlations of TPC with DPPH and hydroxyl radical scavenging activities are consistent with the well‐established ability of plant phenolics to donate hydrogen atoms or electrons and stabilize radical intermediates [27]. The concordant relationships obtained from PLSR and Spearman’s analyses provide mutually supportive evidence of a strong association between TPC and the radical scavenging responses of the investigated *Pandanus* extracts. TFC showed only moderate correlations with the three bioactivities, despite the high flavonoid contents observed in several *P. tectorius* samples. This may reflect the structural diversity of flavonoids because their biological activities depend on the number and position of hydroxyl groups, glycosylation pattern, degree of oxidation, and other structural features rather than on total concentration alone [27, 28]. The exceptionally strong association between TSC and *α*‐amylase inhibition represents one of the most important outcomes of this study. Saponins from medicinal plants have been widely discussed as potential antidiabetic agents, with reported effects on carbohydrate digestion, glucose uptake, insulin signaling, and other metabolic pathways [29]. Flavonoids are also capable of inhibiting *α*‐amylase and *α*‐glucosidase, although their potency is highly structure‐dependent [28]. The present results indicate that TSC was strongly associated with *α*‐amylase inhibition in *Pandanus*, while phenolic and flavonoid contents showed additional, but more moderate, associations with this response. This interpretation is compatible with the relatively high *α*‐amylase inhibition observed in the saponin‐rich extracts of *P. tectorius* and *P. tonkinensis*. However, because no purified saponins were directly tested, the correlation cannot establish a causal relationship. TSC may also covary with unidentified compounds that are the actual enzyme inhibitors. Bioactivity‐guided fractionation and direct testing of isolated constituents will therefore be necessary to determine whether saponins themselves account for the observed association.

Because the present study examined crude extracts using colorimetric assays, nonspecific interactions between phenolic‐rich extracts and enzyme proteins, as well as possible optical interference from colored extract constituents, cannot be completely excluded. Accordingly, the observed phytochemical–bioactivity associations should be interpreted cautiously. Appropriate interference controls, orthogonal assay formats, enzyme‐kinetic experiments, bioactivity‐guided fractionation, and direct testing of isolated constituents will be required to verify the compounds associated with the observed enzyme inhibition. The interpretation of the multivariate results also requires some caution. Because the data were pooled across species and plant organs, the very strong correlations may partly reflect separation between chemically distinct taxa, particularly the phenolic‐rich *P. tectorius* and alkaloid‐rich *P. amaryllifolius*, rather than exclusively representing relationships within each species. The unequal number of samples among species, especially the limited representation of *P. humilis* and *P. tonkinensis*, should also be considered. Future studies should include more balanced biological replication, repeated sampling across seasons and locations, and statistical models that distinguish interspecific from intraspecific effects. Despite these limitations, the present investigation advances knowledge of the genus by demonstrating clear phytochemical differentiation and identifying corresponding bioactivity patterns under standardized experimental conditions. *P. tectorius* can be prioritized as a source of phenolic‐rich antioxidant and *α*‐amylase‐inhibitory extracts, while *P. tonkinensis* merits further investigation for potentially selective enzyme‐inhibitory constituents. The alkaloid‐rich profile of *P. amaryllifolius* supports its evaluation in biological models more closely related to its previously reported anti‐inflammatory and antimicrobial properties. Comprehensive LC–MS/MS profiling, quantitative analysis of individual marker compounds, bioactivity‐guided isolation, enzyme‐kinetic studies, and in vivo validation will be essential to investigate the constituent–activity associations observed here at the compound level. Thus, the principal novelty of this study lies not only in the inclusion of several poorly studied Vietnamese *Pandanus* species but also in the integrated comparative framework used to identify species‐ and metabolite‐class‐specific bioactivity patterns.

## 4. Conclusion

In summary, this study provides a comparative assessment of phytochemical composition and bioactivities across seven *Pandanus* species from Vietnam, revealing pronounced interspecific variation together with selected exploratory organ‐related patterns. *P. tectorius* was characterized by high phenolic, flavonoid, and saponin contents and comparatively strong radical scavenging and *α*‐amylase‐inhibitory activities, whereas the other species exhibited distinct phytochemical and bioactivity profiles. Integrated Spearman’s correlation and PLS regression analyses indicated that phenolic content was most strongly associated with free radical scavenging activity, whereas saponin content showed the strongest association with *α*‐amylase inhibition. These findings expand current knowledge of phytochemical–bioactivity patterns within the genus *Pandanus* and provide a rational basis for the targeted investigation of underexplored species in future phytochemical and pharmacological studies.

## Author Contributions

Do Hoang Giang: conceptualization, visualization, and writing–original draft; Le Thanh Huong: investigation and data curation; Hoang Thuy Duong and Nguyen Thu Uyen: investigation; Nguyen Tien Dat: methodology and supervision; Nguyen Hai Dang: Project administration, conceptualization, supervision, and writing–review and editing.

## Funding

This work was supported by the University of Science and Technology of Hanoi (USTH) under the Top‐tier Research Group Phytomed grant.

## Conflicts of Interest

The authors declare no conflicts of interest.

## Supporting Information

Additional supporting information can be found online in the Supporting Information section.

## Supporting information


**Supporting Information** Plant materials. Chemical assays. Table S1. The information on the collected *Pandanus* samples. Table S2. Total phenolic (TPC), flavonoid (TFC), saponin (TSC), and alkaloid (TAC) of methanol extracts from 45 *Pandanus* plant samples. Table S3. PLS regression model performance for predicting bioactivities of *Pandanus* extracts. Table S4. Percentage of *X*‐variance explained by individual PLS components. Table S5. Root mean square error of prediction (RMSEP) of the PLS regression models. Table S6. Variable importance in projection (VIP) scores of phytochemical variables in the PLS regression model. Table S7. Spearman’s rank correlation coefficients (*ρ*) between phytochemical constituents and bioactivities of *Pandanus* extracts. Table S8. Statistical significance (*p* values) of Spearman’s rank correlation analysis between phytochemical constituents and bioactivities of *Pandanus* extracts.

## Data Availability

All data generated or analyzed during this study are included in this published article and its supporting information files.
