# Supplementary material for: Comparative Phytochemical Profiles and Bioactivity–Constituent Relationships in Seven Pandanus Species From Vietnam
Source: J Anal Methods Chem. 2026 Oct 17;2026:6373690. doi: 10.1155/jamc/6373690 (PMC13676034; doi:10.1155/jamc/6373690)
Supplement: Supplementary file 1 [file JAMC-2026-6373690-s001.docx]

**SUPPLEMENTAL**

# Plant materials

Species names, parts, codes, number of samples, times, and locations of *Pandanus* samples are presented in Table S1. These were identified by Dr. Bui Van Thanh, Institute of Biology, Vietnam Academy of Science and Technology. Voucher specimens of the plants were deposited at the High Technology Innovation Center, Vietnam Academy of Science and Technology.

Table S1. The information on the collected Pandanus samples

| **No.** | **Species** | **Voucher IDs** | **Part** | **Code** | **Number of samples** | **Time** | **Coordinates** |
| --- | --- | --- | --- | --- | --- | --- | --- |
| 1 | *Pandanus tectorius* | NCCG 200105, NCCG 240116, NCCG 240117 | Leaves | Ptec 01-03 | 3 | Feb. 2020 | 20.912181 °N, 105.737122 °E |
|  |  |  |  | Ptec 04-05 | 2 | Mar. 2021 |  |
|  |  |  |  | Ptec 06 | 1 | Jan. 2024 | 22.407260 °N, 105.438909 °E |
|  |  |  |  | Ptec 07 | 1 | Aug. 2024 | 19.162208 °N, 105.730549 °E |
|  |  |  | Fruits | Ptec 08-09 | 2 | Feb. 2020 | 20.912181 °N, 105.737122 °E |
|  |  |  |  | Ptec 10-11 | 2 | Mar. 2021 |  |
|  |  |  |  | Ptec 12 | 1 | Jan. 2024 | 22.407260 °N, 105.438909 °E |
| 2 | *Pandanus amaryllifolius* | NCCG 21013, NCCG 230205 | Leaves | Pama 01-03 | 3 | Apr. 2021 | 21.425607 °N, 105.615562 °E |
|  |  |  |  | Pama 04 | 1 | May. 2023 | 21.05096 °N,  105.23032 °E |
|  |  |  |  | Pama 05 | 1 | Mar. 2024 |  |
|  |  |  |  | Pama 06 | 1 | Jul. 2024 | 21.05100 °N,  105.23031 °E |
| 3 | *Pandanus odoratissimus* | NCCG 210209, NCCG 240410 | Leaves | Podo 01-03 | 3 | May 2021 | 11.99056 °N,  109.20935 °E |
|  |  |  |  | Podo 04-05 | 2 | Jun. 2024 |  |
|  |  |  |  | Podo 06 | 1 | Oct. 2024 | 19.10627 °N, 105.73690 °E |
|  |  |  | Stems | Podo 07-09 | 3 | May 2021 | 11.99056 °N,  109.20935 °E |
|  |  |  |  | Podo 10-11 | 2 | Jun. 2024 |  |
|  |  |  |  | Podo 12 | 1 | Oct. 2024 | 19.10627 °N, 105.73690 °E |
| 4 | *Pandanus affinis* | NCCG 200116 | Leaves | Paff 01-02 | 2 | March 2020 | 15.69166 °N, 108.16504 °E |
|  |  |  | Stems | Paff 03-04 | 2 |  |  |
|  |  |  | Fruits | Paff 05 | 1 |  |  |
| 5 | *Pandanus nanofrutex* | NCCG 200115 | Leaves | Pnano 01 | 1 | Mar. 2020 | 15.68729 °N, 108.15948 °E |
|  |  |  |  | Pnano 02-03 | 2 | Nov. 2021 |  |
|  |  |  | Stems | Pnano 04 | 1 | Mar. 2020 |  |
|  |  |  |  | Pnano 05-06 | 2 | Nov. 2021 |  |
| 6 | *Pandanus humilis* | NCCG 220101 | Leaves | Phum 01-02 | 2 | Nov. 2021 | 15.69266 °N, 108.13783 °E |
| 7 | *Pandanus tonkinensis* | NCCG 220102 | Leaves | Ptonk 01-02 | 2 | Apr. 2022 | 16.17818 °N, 107.63130 °E |
| **Total** | | | | | **45** |  |  |

The plant organs collected differed among species because of species-specific morphological characteristics and the availability or prevalence of particular organs at the sampling sites and times. Consequently, not all plant organs were available for collection from every species, resulting in an unequal representation of leaves, stems, and fruits across the dataset.

# Chemical assays

***Total phenolic assay***

The total phenolic contents of the samples were evaluated by Folin-Ciocalteu method ^1^. The dried methanolic extracts prepared as described in the Sample preparation section were redissolved in methanol at an appropriate concentration and used directly for the assay. Gallic acid solutions at concentrations ranging from 10 to 400 µg/mL were used to construct the external calibration curve. An aliquot (100 µL) of each extract or standard solution was mixed with 400 µL of 10% Folin–Ciocalteu reagent and 500 µL of 6% Na₂CO₃ solution. The mixture was incubated at 40°C for 15 min, and its absorbance was measured at 750 nm using a UV–Vis spectrophotometer. All measurements were performed in triplicate. TPC was calculated from the gallic acid calibration curve and expressed as milligrams of gallic acid equivalents per gram of dried extract (mg GAE/g extract).

***Total flavonoid assay***

The total flavonoid contents of the samples were examined using a previous method ^1^. The dried methanolic extracts prepared as described in the Sample preparation section were redissolved in methanol at an appropriate concentration and used directly for the assay. Quercetin solutions at concentrations ranging from 10 to 200 µg/mL were used to construct the external calibration curve. Briefly, 500 µL of each extract or standard solution was mixed with 100 µL of 5% NaNO₂ solution and maintained at room temperature for 5 min. Subsequently, 100 µL of 10% AlCl₃ solution and 500 µL of 1 M NaOH solution were added. After incubation at room temperature for 15 min, the absorbance was measured at 510 nm using a UV–Vis spectrophotometer. All measurements were performed in triplicate. TFC was calculated from the quercetin calibration curve and expressed as milligrams of quercetin equivalents per gram of dried extract (mg QE/g extract).

***Total alkaloid assay***

The total alkaloid contents of the samples were quantified by a previous method.^2^. The dried methanolic extracts prepared as described in the Sample preparation section were used directly for alkaloid enrichment. Briefly, 5 g of each dried extract was suspended in 50 mL of distilled water and acidified to pH 2 with 1 M HCl. The acidified suspension was partitioned three times with ethyl acetate (3 × 75 mL), and the ethyl acetate layers were discarded. The remaining aqueous phase was adjusted to pH 11 with 1 M NaOH and subsequently extracted four times with CH₂Cl₂ (4 × 200 mL). The CH₂Cl₂ layers were combined and evaporated under reduced pressure to obtain the total alkaloid fraction. TAC was determined gravimetrically and expressed as the percentage by weight of the dried methanolic extract (% w/w).

***Total saponin assay***

The total saponin contents (TSC) of the samples were determined by vanillin-sulphuric acid assay ^3^. The dried methanolic extracts prepared as described in the Sample preparation section were redissolved in methanol at an appropriate concentration and used directly for the assay. Briefly, 50 µL of each extract solution was mixed with 50 µL of 8% (w/v) vanillin in ethanol and 1,500 µL of 72% (v/v) sulfuric acid. The mixture was incubated at 70°C for 15 min and subsequently cooled at room temperature for 5 min. Aescin solutions at different concentrations were used to construct the external calibration curve, while the corresponding solvent was used as the reagent blank. The absorbance was measured at 560 nm against the blank using a UV–Vis spectrophotometer. All measurements were performed in triplicate. TSC was calculated from the aescin calibration curve and expressed as milligrams of aescin equivalents per gram of dried extract (mg AE/g extract).

## Bioassays

***Antioxidant assays***

The antioxidant potential of the extracts was assessed using DPPH and hydroxyl radical scavenging assays, following established protocols ^4-5^.

For the DPPH assay, 100 µL of each sample was mixed with 1900 µL of DPPH solution in methanol and incubated in the dark at 37°C for 20 minutes. Absorbance was measured at 517 nm, with ascorbic acid serving as the reference standard.

In the hydroxyl radical scavenging assay, a 200 µL aliquot of each test sample was added to a mixture containing 400 µL of 50 mM phosphate buffer (pH 7.8), 400 µL of 2.8 mM deoxyribose, and 400 µL of 500 µM ferrous ammonium sulfate [Fe(NH_4_)_2_(SO_4_)_2_]. The mixture was incubated at 37°C for 1 hour. The reaction was terminated by the addition of 1000 µL of 10% (w/v) trichloroacetic acid and 1000 µL of 1% (w/v) thiobarbituric acid. The resulting solution was then heated in a boiling water bath for 15 minutes, and absorbance was measured at 532 nm. Catechin was used as the positive control for this assay.

***α-amylase inhibitory activity***

The *α*-amylase inhibitory activity was determined using a modified version of the DNSA method ^6^. The substrate was prepared by boiling 1 g of starch in 100 mL of phosphate buffer (pH 7.0) for 10 minutes and then allowed to cool to room temperature. The DNSA reagent was prepared by dissolving 12 g of sodium potassium tartrate tetrahydrate in 8 mL of 2 M NaOH and 20 mL of 96 mM 3,5-dinitrosalicylic acid solution. The extracts were initially dissolved in 10% DMSO, followed by further dilution in buffer (Na_2_HPO_4_/NaH_2_PO_4_ (0.02 M), NaCl (0.006 M), pH 6.9) to achieve various concentrations. A 200 µL aliquot of each sample solution was mixed with 200 µL of *α*-amylase solution (2 units/mL) and incubated at 37°C for 3 minutes. Subsequently, 200 µL of the starch substrate was added, and the reaction mixture was incubated at 37°C for 15 minutes. The reaction was stopped by adding 200 µL of the DNSA reagent, and the solution was boiled for 10 minutes in a water bath at 85–90°C. After cooling to room temperature, the absorbance was measured at 650 nm using a microplate reader, with acarbose serving as the positive control.

*Table S2. Total phenolic (TPC), flavonoid (TFC), saponin (TSC), and alkaloid (TAC) of methanol extracts from 45 Pandanus plant samples*

| **No.** | **Type** | **Part** | **Code** | **TPC**  **(mgGAE/g)** | **TFC**  **(mgQE/g)** | **TSC**  **(mgAE/g)** | **TAC**  **(%)** |
| --- | --- | --- | --- | --- | --- | --- | --- |
| 1 | *P. tectorius* | Leaves | Ptec01 | 82.86 ± 13.58 | 34.78 ± 5.35 | 29.13 ± 1.45 | 1.21 ± 0.25 |
| 2 | *P. tectorius* | Leaves | Ptec02 | 78.82 ± 13.53 | 35.81 ± 6.84 | 31.46 ± 3.49 | 1.15 ± 0.15 |
| 3 | *P. tectorius* | Leaves | Ptec03 | 88.18 ± 12.58 | 34.24 ± 7.14 | 29.19 ± 2.51 | 1.25 ± 0.16 |
| 4 | *P. tectorius* | Leaves | Ptec04 | 86.32 ± 13.56 | 33.2 ± 3.46 | 27.04 ± 3.46 | 1.27 ± 0.28 |
| 5 | *P. tectorius* | Leaves | Ptec05 | 70.41 ± 10.88 | 29.03 ± 4.46 | 24.13 ± 1.23 | ND |
| 6 | *P. tectorius* | Leaves | Ptec06 | 63.14 ± 10.50 | 27.65 ± 5.05 | 22.91 ± 2.16 | ND |
| 7 | *P. tectorius* | Leaves | Ptec07 | 115.71 ± 8.96 | 39.85 ± 2.13 | 48.93 ± 3.85 | ND |
| 8 | *P. tectorius* | Fruits | Ptec08 | 127.26 ± 9.39 | 36.48 ± 1.97 | 52.59 ± 4.8 | ND |
| 9 | *P. tectorius* | Fruits | Ptec09 | 105.68 ± 9.44 | 38.2 ± 2.18 | 47.61 ± 2.62 | ND |
| 10 | *P. tectorius* | Fruits | Ptec10 | 114.03 ± 8.32 | 36.37 ± 2.32 | 51.82 ± 1.67 | ND |
| 11 | *P. tectorius* | Fruits | Ptec11 | 93.29 ± 7.42 | 31.49 ± 1.77 | 38.83 ± 4.24 | ND |
| 12 | *P. tectorius* | Fruits | Ptec12 | 92.04 ± 6.97 | 31.07 ± 1.7 | 38.73 ± 3.02 | ND |
| 13 | *P. amaryllifolius* | Leaves | Pama01 | 42.53 ± 5.45 | 5.27 ± 0.43 | 7.54 ± 0.69 | 15.11 ± 0.79 |
| 14 | *P. amaryllifolius* | Leaves | Pama02 | 45.46 ± 5.22 | 6.57 ± 0.47 | 8.58 ± 0.58 | 8.68 ± 0.72 |
| 15 | *P. amaryllifolius* | Leaves | Pama03 | 52.07 ± 4.94 | 6.97 ± 0.64 | 9.88 ± 0.75 | 21.76 ± 0.93 |
| 16 | *P. amaryllifolius* | Leaves | Pama04 | 37.92 ± 8.05 | 4.36 ± 0.38 | 11.12 ± 0.75 | 21.14 ± 1.12 |
| 17 | *P. amaryllifolius* | Leaves | Pama05 | 46.38 ± 7.64 | 5.78 ± 0.39 | 8.52 ± 0.96 | 16.57 ± 1.04 |
| 18 | *P. amaryllifolius* | Leaves | Pama06 | 34.62 ± 4.96 | 6.92 ± 0.36 | 8.13 ± 1.01 | 17.01 ± 1.11 |
| 19 | *P. odoratissimus* | Leaves | Podo01 | 51.21 ± 7.72 | 29.27 ± 3.02 | 19.96 ± 1.24 | ND |
| 20 | *P. odoratissimus* | Leaves | Podo02 | 71.68 ± 9.58 | 25.57 ± 4.22 | 20.42 ± 1.83 | ND |
| 21 | *P. odoratissimus* | Leaves | Podo03 | 58.39 ± 7.02 | 21.64 ± 3.92 | 18.41 ± 1.56 | ND |
| 22 | *P. odoratissimus* | Leaves | Podo04 | 61.79 ± 6.93 | 21.72 ± 3.87 | 18.1 ± 1.63 | ND |
| 23 | *P. odoratissimus* | Leaves | Podo05 | 56.62 ± 6.22 | 18.69 ± 3.58 | 17.36 ± 1.41 | ND |
| 24 | *P. odoratissimus* | Leaves | Podo06 | 54.23 ± 6.83 | 20.59 ± 2.86 | 14.59 ± 1.54 | ND |
| 25 | *P. odoratissimus* | Stems | Podo07 | 76.78 ± 8.31 | 8.96 ± 1.11 | 23.23 ± 1.34 | ND |
| 26 | *P. odoratissimus* | Stems | Podo08 | 43.21 ± 8.72 | 6.31 ± 0.84 | 21.61 ± 1.08 | ND |
| 27 | *P. odoratissimus* | Stems | Podo09 | 60.11 ± 9.46 | 5.42 ± 0.55 | 28.13 ± 1.21 | ND |
| 28 | *P. odoratissimus* | Stems | Podo10 | 46.58 ± 7.84 | 6.53 ± 0.27 | 27.03 ± 0.9 | ND |
| 29 | *P. odoratissimus* | Stems | Podo11 | 46.21 ± 9.35 | 3.21 ± 0.19 | 27.95 ± 1.02 | ND |
| 30 | *P. odoratissimus* | Stems | Podo12 | 37.01 ± 8.41 | 9.99 ± 1.45 | 39.25 ± 0.96 | ND |
| 31 | *P. affinis* | Leaves | Paff01 | 36.26 ± 5.67 | 14.95 ± 1.74 | 14.95 ± 1.31 | ND |
| 32 | *P. affinis* | Leaves | Paff02 | 46.28 ± 7.29 | 13.98 ± 2.25 | 18.37 ± 1.8 | ND |
| 33 | *P. affinis* | Stems | Paff03 | 24.23 ± 2.87 | 17.79 ± 1.38 | 2.27 ± 0.13 | ND |
| 34 | *P. affinis* | Stems | Paff04 | 22.79 ± 2.83 | 20.15 ± 1.54 | 2.77 ± 0.17 | ND |
| 35 | *P. affinis* | Fruits | Paff05 | 41.98 ± 4.34 | 27.81 ± 2.23 | 3.45 ± 0.21 | ND |
| 36 | *P. nanofrutex* | Leaves | Pnano01 | 21.32 ± 1.85 | 12.17 ± 1.01 | 17.78 ± 0.83 | ND |
| 37 | *P. nanofrutex* | Leaves | Pnano02 | 23.04 ± 1.76 | 10.71 ± 0.91 | 20.49 ± 1.01 | ND |
| 38 | *P. nanofrutex* | Leaves | Pnano03 | 31.29 ± 1.91 | 13.52 ± 1.47 | 21.95 ± 1.21 | ND |
| 39 | *P. nanofrutex* | Stems | Pnano04 | 19.18 ± 1.68 | 10.81 ± 0.88 | 15.89 ± 0.76 | ND |
| 40 | *P. nanofrutex* | Stems | Pnano05 | 19.68 ± 1.56 | 9.77 ± 0.88 | 16.54 ± 0.71 | ND |
| 41 | *P. nanofrutex* | Stems | Pnano06 | 18.42 ± 1.52 | 10.02 ± 0.89 | 14.45 ± 0.68 | ND |
| 42 | *P. humilis* | Leaves | Phum01 | 15.95 ± 2.43 | 9.14 ± 0.82 | 24.03 ± 2.03 | ND |
| 43 | *P. humilis* | Leaves | Phum02 | 13.06 ± 2.43 | 13.52 ± 0.74 | 23.03 ± 2.65 | ND |
| 44 | *P. tonkinensis* | Leaves | Ptonk01 | 25.98 ± 3.91 | 21.68 ± 2.86 | 30.79 ± 1.75 | ND |
| 45 | *P. tonkinensis* | Leaves | Ptonk02 | 24.86 ± 3.87 | 23.49 ± 3.02 | 25.64 ± 1.68 | ND |
| **ND**: not detected (below the limit of detection) | | | | | | | |

*Table S3. PLS regression model performance for predicting bioactivities of Pandanus extracts*

| **opt_ncomp** | **R^2^X_train_cum** | **Q^2^_mean** |
| --- | --- | --- |
| 4 | 1 | 0.888948892 |
| **Response** | **R^2^Y_train** | **Q^2^_CV** |
| DPPH | 0.913788652 | 0.885556628 |
| OH | 0.917037959 | 0.902073222 |
| alpha_amylase | 0.897882515 | 0.879216827 |

The table summarizes the optimal number of components (opt_ncomp), cumulative explained variance of the X-matrix (R²X_train_cum), mean cross-validated predictive ability (Q²_mean), coefficient of determination for calibration (R²Y_train) and cross-validation (Q²_CV) of the PLS regression models established between phytochemical contents (TPC, TFC, TSC, and TAC) and the bioactivities, including DPPH radical scavenging, hydroxyl radical scavenging (OH), and α-amylase inhibition.

*Table S4. Percentage of X-variance explained by individual PLS components.*

| **Component** | **X_var (%)** | **X_var_cum (%)** |
| --- | --- | --- |
| Comp1 | 63.15 | 63.15 |
| Comp2 | 22.41 | 85.56 |
| Comp3 | 10.13 | 95.69 |
| Comp4 | 4.31 | 100.00 |

The contribution of each PLS component (Comp1–Comp4) to the explained variance of the X-matrix (phytochemical variables) is presented as individual (X_var) and cumulative (X_var_cum) percentages.

*Table S5. Root mean square error of prediction (RMSEP) of the PLS regression models.*

| **Response** | **RMSEP_train** | **RMSEP_CV** |
| --- | --- | --- |
| DPPH | 0.052333 | 0.060296 |
| OH | 0.052348 | 0.056874 |
| alpha_amylase | 0.053134 | 0.057786 |

RMSEP values for calibration (RMSEP_train) and cross-validation (RMSEP_CV) are reported for each bioactivity response, reflecting the predictive accuracy and robustness of the PLS models.

*Table S6. Variable importance in projection (VIP) scores of phytochemical variables in the PLS regression model.*

| **Variable** | TPC | TSC | TFC | TAC |
| --- | --- | --- | --- | --- |
| **VIP** | 1.26527 | 1.02759 | 0.95534 | 0.65611 |

VIP scores indicate the relative contribution of total phenolic content (TPC), total saponin content (TSC), total flavonoid content (TFC), and total alkaloid content (TAC) to the prediction of bioactivities. Variables with VIP values above 1 were considered to have comparatively high predictive importance in the model.

*Table S7. Spearman’s rank correlation coefficients (ρ) between phytochemical constituents and bioactivities of Pandanus extracts.*

|  | **Spearman_ρ** | | |
| --- | --- | --- | --- |
|  | **DPPH** | **OH** | **alpha_amylase** |
| **TPC** | 0.98310 | 0.98696 | 0.65599 |
| **TFC** | 0.55972 | 0.57051 | 0.54514 |
| **TSC** | 0.54198 | 0.57931 | 0.97852 |
| **TAC** | 0.12057 | 0.13813 | -0.24331 |

The table presents Spearman’s correlation coefficients (ρ) describing the monotonic relationships between phytochemical parameters, including total phenolic content (TPC), total flavonoid content (TFC), total saponin content (TSC), and total alkaloid content (TAC), and the bioactivities of *Pandanus* extracts, namely DPPH radical scavenging, hydroxyl radical scavenging (OH), and α-amylase inhibitory activity. ρ values close to ±1 indicate strong monotonic correlations

*Table S8. Statistical significance (p-values) of Spearman’s rank correlation analysis between phytochemical constituents and bioactivities of Pandanus extracts*

| **Variable** | **Spearman_p values** | | |
| --- | --- | --- | --- |
|  | **DPPH** | **OH** | **α-amylase** |
| TPC | < 0.001 | < 0.001 | < 0.001 |
| TFC | < 0.001 | < 0.001 | < 0.001 |
| TSC | < 0.001 | < 0.001 | < 0.001 |
| TAC | 0.43 | 0.366 | 0.107 |

Corresponding p-values for Spearman’s rank correlation coefficients (ρ) are reported to assess the statistical significance of the associations between phytochemical variables (TPC, TFC, TSC, TAC) and biological activities (DPPH, OH, and α-amylase inhibition). p-values < 0.05 were considered statistically significant.

**REFERENCE**

1.	Fattahi, S.; Zabihi, E.; Abedian, Z.; Pourbagher, R.; Motevalizadeh Ardekani, A.; Mostafazadeh, A.; Akhavan-Niaki, H., Total Phenolic and Flavonoid Contents of Aqueous Extract of Stinging Nettle and In Vitro Antiproliferative Effect on Hela and BT-474 Cell Lines. *International journal of molecular and cellular medicine* **2014,** *3* (2), 102-7.

2.	Vietnam, M. o. H. o., *Vietnamese Pharmacopoeia V*. Medical Publishing House: Ha Noi, Vietnam, 2017.

3.	Le, A. V.; Parks, S. E.; Nguyen, M. H.; Roach, P. D., Improving the Vanillin-Sulphuric Acid Method for Quantifying Total Saponins. *Technologies* **2018,** *6* (3), art.ID. 84.

4.	Brand-Williams, W.; Cuvelier, M. E.; Berset, C., Use of a free radical method to evaluate antioxidant activity. *LWT - Food Science and Technology* **1995,** *28* (1), 25-30.

5.	Thuong, P. T.; Su, N. D.; Ngoc, T. M.; Hung, T. M.; Dang, N. H.; Thuan, N. D.; Bae, K. H.; Oh, W. K., Antioxidant activity and principles of Vietnam bitter tea *Ilex kudingcha*. *Food Chemistry* **2009,** *113* (1), 139-145.

6.	Sudha, P.; Zinjarde, S. S.; Bhargava, S. Y.; Kumar, A. R., Potent α-amylase inhibitory activity of Indian Ayurvedic medicinal plants. *BMC Complementary and Alternative Medicine* **2011,** *11* (1), 5.
